# Supplementary material for: Study on surface deformation induced by shield excavation due to coarse particle content in strongly weathered rock layers
Source: PLoS One. 2025 Apr 23;20(4):e0321034. doi: 10.1371/journal.pone.0321034 (PMC12017534; doi:10.1371/journal.pone.0321034)
Supplement: Attachment — Submitted filename: Response to Reviewers2.doc [file pone.0321034.s002.doc]

Dear professor Paula Katrina A. Maderazo and reviewers,

We’re very glad to hear from you, we have carefully addressed each comment and corrected it as needed. The modified part is marked red on the paper. Detailed responses to reviewer comments are as follows:

1. We note that your Data Availability Statement is currently as follows: [All relevant data are within the manuscript and its Supporting Information files.]Please confirm at this time whether or not your submission contains all raw data required to replicate the results of your study. Authors must share the “minimal data set” for their submission. PLOS defines the minimal data set to consist of the data required to replicate all study findings reported in the article, as well as related metadata and methods

Reply: I have confirmed that the content submitted includes all the original data necessary to replicate the research results.

2. we note that your Response to Reviewers and Track Changes files appears to highlight only changes made in response to the editorial comments and not the comments provided by reviewers. Please therefore provide an updated Response to Reviewers file that includes a point-by-point response to each of the reviewers' comments, and an updated Track Changes file that highlights changes made in response to both the editorial comments and reviewer comments.

Reply: An updated "Response to Reviewers" file has been uploaded according to the recommendations, which includes a point-by-point response to each reviewer's comments and provides a new "Tracked Changes" file.
