# Supplementary material for: Study on surface deformation induced by shield excavation due to coarse particle content in strongly weathered rock layers
Source: PLoS One. 2025 Apr 23;20(4):e0321034. doi: 10.1371/journal.pone.0321034 (PMC12017534; doi:10.1371/journal.pone.0321034)
Supplement: Attachment — Submitted filename: Response to Reviewers.docx [file pone.0321034.s003.docx]

**Responses to Editor and Reviewers**

Dear editorial board members,

We have addressed each comment carefully and made corrections as needed. The revised portions are marked in red in the paper. The response to the editorial follows:

(1) We note that Reviewer 3 recommended that you cite specific previously published works in an earlier round of revision. As always, we recommend that you please review and evaluate the requested works to determine whether they are relevant and should be cited. It is not a requirement to cite these works and you may remove them before the manuscript proceeds to publication. We appreciate your attention to this request.

Response: Thanks to your reminder, I have scrutinized the references in the article, reviewing and evaluating the references cited and removing those of low relevance to the topic of the article.

Revised contents:

Delete:28.	Gong C, Cheng M, Peng Y, et al. Seepage propagation simulation of a tunnel gasketed joint using the cohesive zone model[J]. Tunnelling and Underground Space Technology, 2024, 147: 105726.

https://doi.org/10.1016/j.tust.2024.105726

29.	Gong C, Xie C, Zhu H, et al. Time-varying compressive properties and constitutive model of EPDM rubber materials for tunnel gasketed joint[J]. Construction and Building Materials, 2024, 433: 136734. <https://doi.org/10.1016/j.conbuildmat.2024.136734>

(2) Please review your reference list to ensure that it is complete and correct. If you have cited papers that have been retracted, please include the rationale for doing so in the manuscript text, or remove these references and replace them with relevant current references. Any changes to the reference list should be mentioned in the rebuttal letter that accompanies your revised manuscript. If you need to cite a retracted article, indicate the article’s retracted status in the References list and also include a citation and full reference for the retraction notice.

Response: Thank you for the correction, I have double checked the reference list for completeness and accuracy and have not cited withdrawn papers.

Revised contents:

1. Yi G L, Chen K, Lu G M, et al. Progress in shield tunneling technology for urban underground space in China [J]. Tunnel Construction, 2024,44(01):1-20. https:// doi.org/10.3973/j.issn.2096-4498.2024.01.001
2. Peck B B. Deep excavation and tunnelling in soft ground, State of the art volume[J].Icsmfe, 1969, 4: 225-290
3. Song X H. Ground surface settlement monitoring and analysis on the deformation prediction by Peck formula in shield tunnel construction [J]. Railway Construction Technology, 2021,(6): 11-16. https://doi.org/10.3969/j.issn.1009-4539.2021.06.003
4. Yan S H, Wen C P. Application of modified peck formula in surface settlement of hejiapo tunnel, PREPRINT (Version 1) available at Research Square, 2023, http:// doi.org/10.21203/rs.3.rs-2032575/v1
5. Wang S Y, Song Z P, Tian X X, et al. Prediction for the surface settlement of double-track subway tunnels for shallow buried loess based on peck formula. Frontiers in Earth Science, 2023, 11: 1219544. http:// doi.org/10.3389/feart. 2023.1219544
6. Yang F F, Yan H B, Tian L, et al. Research on the applicability of Peck modified formula in surface subsidence prediction of shield tunnel with water-rich sand and pebble formation[J]. Geotechnical Investigation & Surveying, 2021, 49(11): 72-78
7. Zhang Q, Wu K, Cui S, et al. Surface settlement induced by subway tunnel construction based on modified peck formula[J]. Geotechnical and Geological Engineering, 2019, 37: 2823-2835. http://doi.org/10.1007/s10706-018-00798-6
8. Yin G M, Fu H L, Hou W Z, et al. Introduction of several methods of value of Peck formula parameters[J/OL]. Journal of Railway Science and Engineering, 2022, 19(7): 2015-2022. http:// doi.org/10.19713/j.cnki.43-1423/u.T20210810
9. Zhang Z, Chen J, Zhang M, et al. Modified Peck method of evaluating tunnelling-induced ground movements based on measured data[J]. Arabian Journal of Geosciences, 2022, 15(9): 862. https://doi.org/10.1007/s12517-022-10142-1
10. Huang R, Zhu J X, Zhu X J, et al. Analysis of Ground Settlement during Shield Tunnel Construction in Soft Soil[J]. Soil Mechanics and Foundation Engineering, 2023, 60(2): 134-140. https://doi.org/10.1007/s11204-023-09874-3
11. Liu J J, Liu J W, Ren X M, et al. Modification of Peck formula for ground setllement caused by shield tunneling in soil-rock composite stratum [J/OL]. Journal of Henai Polytechnic University (Natural Science), 2022, 41(2): 171-177. http://doi.org/10.16186/j.cnki.1673-9787.2020060011.
12. Wang F, Du X, Li P. Predictions of ground surface settlement for shield tunnels in sandy cobble stratum based on stochastic medium theory and empirical formulas[J]. Underground Space, 2023, 11: 189-203. https://doi.org/10.1016/j.undsp.2023.01.003
13. Wei G, Zhang X, Xu Y, et al. Prediction of ground settlement due to excavation of a quasi-rectangular shield tunnel based on stochastic medium theory[J]. Geotechnical and Geological Engineering, 2019, 37: 3605-3618. https://doi.org/10.1007/s10706-019-00856-7
14. Shao Z S, Zhao X. Based on extension of stochastic medium theory prediction model for surface subsidence induced by tunnel construction[J/OL]. Journal of Chang'an University(Natural Science Edition), 2021, 41(6): 73-81. http:// doi.org/10.19721/j.cnki.1671-8879.2021.06.008
15. Zeng B, Huang D. Soil deformation induced by Double-O-Tube shield tunneling with rolling based on stochastic medium theory[J]. Tunnelling and Underground Space Technology, 2016, 60: 165-177. https://doi.org/10.1016/j.tust.2016.09.001
16. Liu B, Yang W H, Zhang G, et al. A prediction model based on stochastic medium theory for ground surface settlement induced by non-uniform tunnel deformation[J]. Chinese Journal of Rock Mechanics and Engineering, 2018, 37(08) :1943-1952. http:// doi.org/10.13722/j.cnki.jrme.2017.1531
17. Yang J S, Liu B C, Wang M C. Modeling of tunneling-induced ground surface movements using stochastic medium theory[J]. Tunnelling and Underground Space Technology, 2004, 19(2): 113-123. https://doi.org/10.1016/j.tust.2003.07.002
18. Wang T Z. Study on ground deformation caused by subway tunnel construction in Changchun soft mudstone formation[D]. Jilin University, 2017.
19. Di Q, Li P, Zhang M. Experimental study of face stability for shield tunnels in sandy cobble strata of different densities. Tunnelling and Underground Space Technology. 2023, 135. 105029. http://doi.org/10.1016/j.tust.2023.105029.
20. Cai B H, Cui D S, Feng X L, et al. Study on surface deformation pattern caused by small shield tunneling in complex strata in karst area of Wuhan[J]. Safety and Environmental Engineering, 2020, 27(01): 69-74. <http://doi.org/10.13578/j.cnki.issn.>1671-1556. 2020. 01.011.
21. Melis M, Medina L, Rodríguez J M. Prediction and analysis of subsidence induced by shield tunnelling in the Madrid Metro extension[J]. Canadian Geotechnical Journal, 2002, 39(6): 1273-1287. <https://doi.org/10.1139/t02-073>
22. He S, Li C, Wang D, et al. Surface settlement induced by slurry shield tunnelling in sandy cobble strata—A case study[J]. Indian Geotechnical Journal, 2021, 51(6): 1349-1363. https://doi.org/10.1007/s40098-021-00543-6
23. Sun H, Tang K L, Jin A B, et al. Experimental study on influence of single coarse particle on shear properties of ore-rock particle system[J]. Journal of Zhejiang University(Engineering Science), 2022,56(10):2037-2048. https://doi.org/10.3785/j.issn.1008-973X.2022.10.015
24. Hu P, Chen Z B, Luo X, et al. Large direct shear test study on coarse-grained granite residual soil[J]. Journal of Natural Disasters, 2019, 28(06): 119-126. [http://doi.org/10.13577/j.jnd. 2019.0613](http://doi.org/10.13577/j.jnd.%202019.0613).
25. Zhang W G, Li H R, Wu C Z, et al. Soft computing approach for prediction of surface settlement induced by earth pressure balance shield tunneling[J]. Underground Space, 2021, 6(4): 353-363. https://doi.org/10.1016/j.undsp.2019.12.003
26. Jia B X, Gao Z. Investigating surface deformation caused by excavation of curved shield in upper soft and lower hard soil[J]. Frontiers in Earth Science, 2022, 10: 844969. https://doi.org/10.3389/feart.2022.844969
27. Zhang D M, Ma L X, Zhang J, et al. Ground and tunnel responses induced by partial leakage in saturated clay with anisotropic permeability[J]. Engineering Geology, 2015, 189: 104-115. https://doi.org/10.1016/j.enggeo.2015.02.005
28. Chen Y X, Li X Y, Chen S. Prediction of surface settlement caused by shield construction in tunnel based on Peck formula[J]. Safety and Environmental Engineering, 2023,30(02):79-83+91. http:// doi.org/10.13578/j.cnki.issn.1671-1556.20221343.
29. Zhang J C, Zeng C G, Cui L J, et al. Ground settlement prediction of shallow-buried super-large-diameter shield tunnel based on Loganathan & Poulos and Clough modified formulas[J]. Tunnel Construction, 2022,42(S1):274-280. http://doi.org/10.3973/j.issn.2096-4498.2022.S1.032
30. Verruijt A. A complex variable solution for a deforming circular tunnel in an elastic half‐plane[J]. International Journal for Numerical and Analytical Methods in Geomechanics, 1997, 21(2): 77-89. https://doi.org/10.1002/(SICI)1096-9853(199702)21:2<77::AID-NAG857>3.0.CO;2-M
31. Wang X R, Jiang H J, Zhu K, et al. Research on ground settlement laws of urban subway tunnel construction process based on earth pressure shield[J]. Earth Science, 2019,44(12):4293-4298. https://doi.org/[10.3799/dqkx.2019.269](https://doi.org/10.3799/dqkx.2019.269)
32. Luo J J, Zhang D L, Wang M S, et al. Study on the influence of station subsurface excavation on underground pipelines in Beijing Metro[J]. Journal of the China Railway Society, 2007(05):127-132. 2007(05):127-132.
33. O'reilly M P, New B M. Settlements above tunnels in the United Kingdom-their magnitude and prediction[R]. 1982.
